# Enforced symmetry: the necessity of symmetric waxing and waning

**DOI:** 10.7717/peerj.8011

**Published:** 2019-11-06

**Authors:** Niklas Hohmann, Emilia Jarochowska

**Affiliations:** Friedrich-Alexander Universität Erlangen-Nürnberg, Erlangen, Germany

**Keywords:** Symmetry, Range size, Extinction risk, Ecology, Limit theorems, Diversity, Conditioning, Occupancy, Paleontology, Averaging

## Abstract

A fundamental question in ecology is how the success of a taxon changes through time and what drives this change. This question is commonly approached using trajectories averaged over a group of taxa. Using results from probability theory, we show analytically and using examples that averaged trajectories will be more symmetric as the number of averaged trajectories increases, even if none of the original trajectories they were derived from is symmetric. This effect is not only based on averaging, but also on the introduction of noise and the incorporation of a priori known origination and extinction times. This implies that averaged trajectories are not suitable for deriving information about the processes driving the success of taxa. In particular, symmetric waxing and waning, which is commonly observed and interpreted to be linked to a number of different paleobiological processes, does not allow drawing any conclusions about the nature of the underlying process.

## Introduction

The expansion and demise of a taxon during its lifespan is manifested by the temporal dynamics of its geographic range size, occupancy, or—in the case of taxa above the species level—taxonomic richness. Here we refer to these diverse ways of characterizing taxon presence as measures of eco-evolutionary success (MESs). Individual MESs have typically been considered in separate theoretical and empirical frameworks (Willis, 1922; Raup et al., 1973; Gould, Gilinsky & German, 1987; Ricklefs & Bermingham, 2002; Gaston, 1998; Liow & Stenseth, 2007; Pigot, Owens & Orme, 2012). Nonetheless, a shared set of analytical approaches is commonly employed in tracing the changes in these measures through time, either to test model predictions or in an exploratory way to search for patterns. Studies of individual taxa as well as theoretical considerations indicate that a wide range of patterns of a taxon’s rise and fall are possible and realized, including symmetric, skewed, linear and multi-modal (for example Liow et al.(2010) and Pigot, Owens & Orme (2012)). Yet, simulations and analyses based on large datasets spanning geological timescales reveal that, on average, taxa show a symmetrical pattern of waxing and waning, regardless whether their presence is measured as richness, occupancy, geographic range, or any other aspect (Foote, 2007; Foote et al., 2007; Liow & Stenseth, 2007). If averaged across large datasets, these measures show a monotonic increase from the origination of the taxon to the middle of the taxon’s life span, and a monotonic decrease from the middle of the taxon’s life span to its extinction. This creates a symmetric, hat-shaped trajectory that displays no plateaus and is axis-symmetric with respect to the middle of the life span of the taxon (Jernvall & Fortelius, 2004; Foote et al., 2007; Liow & Stenseth, 2007; Quental & Marshall, 2013). This pattern is independent of the MES and of the corresponding units considered. It emerges, for example, in the number of species or the number of grid cells occupied by a taxon per time unit, and has been used to estimate extinction risk as well as origination and extinction times of taxa (Tietje & Kiessling, 2013; Silvestro et al., 2014). The symmetrical pattern does not follow from any theoretical considerations put forward so far (Liow & Stenseth, 2007). It has been acknowledged that symmetry is the expected outcome of a bounded random walk process (Foote, 2014). Nonetheless, averaged patterns continue to be used in paleontology.

The aim of this paper is (1) to provide a mathematical explanation why the symmetry emerging from averaged trajectories is enforced by the procedures commonly used to generate them and (2) demonstrate why the result of averaging cannot be used to infer on the properties of the processes governing the expansion and demise of taxa. This is because the way these trajectories are created will always lead to an increase in symmetry and make patterns resulting from different processes appear more similar, rendering them indistinguishable. Finally, a tenative solution is proposed here. It involves a measure of asymmetry and a nonparametric test whether two sets of temporal trajectories are generated by the same underlying distribution.

We demonstrate theoretically and through examples that mathematical constraints on transforming and averaging taxon trajectories through time have important implications for what can and cannot be derived from these trajectories in terms of mechanisms and patterns of changes in range size (Willis, 1922; Gaston, 1998; Webb & Gaston, 2000), occupancy, and diversity through time (Gould, Gilinsky & German, 1987; Foote, 2007; Foote et al., 2007; Liow & Stenseth, 2007; Pigot, Owens & Orme, 2012; Plotnick & Wagner, 2018). The constraints apply to all research questions in which symmetric waxing and waning has emerged and served to infer on macroecological patterns. These questions can be broadly divided in the three classes summarized below.

### Temporal dynamics of geographic range size and occupancy

Systematic studies of the geographic expansion of taxa over their lifespan were spurred by Willis (1922), who postulated a constant increase of the taxon’s range size over its duration. This trajectory, known as the age and area hypothesis, has been met with many counterexamples. Among them, the concept of taxon cycle (Dillon, 1966; Ricklefs & Cox, 1972; Ricklefs & Cox, 1978) postulated phases of range expansion, establishment of maximum range, fragmentation due to local extinctions, and a final demise. Neither of these trajectories has been found to hold universally (Gaston, 1998; Pigot, Owens & Orme, 2012). Empirical studies revealed such a variety of temporal trends in geographic range sizes, both within and between taxonomic levels, that Gaston (1998) proposed that each species might show an idiosyncratic trajectory that does not allow to infer on any general pattern. Nonetheless, the duration of a taxon and its geographic range are reciprocally influencing each other, depending on which phase of the taxon’s duration is considered (Foote et al., 2008).

But apparent idiosyncrasy of trajectories of individual taxa need not mean that there are no shared mechanisms determining the relationship between range size and age of a taxon. On the contrary, this is consistent with the trajectories being produced by a stochastic process. It has been shown that a wide range of empirical patterns in the age-area relationships observed in diverse groups of organisms can be produced by a single stochastic process (Pigot, Owens & Orme, 2012). This approach took into account two essential parameters: (1) the relationship between the range size and the probabilities of extinction and origination, and (2) the asymmetry of range division during a speciation event (Gaston, 1998). Both parameters vary between taxa substantially and their variation yields all types of range size-age relationships (Pigot, Owens & Orme, 2012). Moreover, all types of trajectories considered in classifications outlined by Gaston (1998) and Liow & Stenseth (2007) can be produced by one stochastic process and their relative frequencies will vary according to the parameters of speciation dynamics and range area division. Notably, averaged simulated range size trajectories from origin to extinction show a symmetrical waxing and waning pattern even though this pattern is not even produced once as an individual trajectory in the simulation (Pigot, Owens & Orme, 2012). Therefore, the underlying stochastic process cannot be reconstructed from empirical trajectories which it produced.

Although the above discussion focuses on geographic range size, the same considerations are—with respect to average patterns—valid for other measures of geographic distribution, such as latitudinal range and various MESs employed in ecology and palaeobiology (for example  Jernvall & Fortelius, 2004; Raia et al., 2006; Foote, 2007; Foote et al., 2007).

### Temporal dynamics of diversity

As a descriptive pattern, symmetry emerges in compilations of averaged diversity trajectories across various taxonomic and temporal scales (Foote, 2007; Foote et al., 2007; Quental & Marshall, 2013). It is also manifested in the averaged CG (center of gravity) measure introduced by Gould, Gilinsky & German (1987), which corresponds to the center of mass of the diversity of a taxon plotted against its relative lifespan. For most of the Phanerozoic and when averaged across multiple taxa, the CG metric is indistinguishable from 0.5 (Gould, Gilinsky & German, 1987; Plotnick & Wagner, 2018), in concordance with symmetric waxing and waning.

When phylogenetic relationships are not known, patterns of diversity consistent with those observed in the fossil record are modeled as a random walk (Cornette & Lieberman, 2004; Žliobaite, Fortelius & Stenseth, 2017). Evolutionary relationships can be represented using branching (or birth-death) processes, which also allow reproducing empirical patterns (Raup et al., 1973; Gould, Gilinsky & German, 1987; Nee, 2006; Foote, 2007; Quental & Marshall, 2009). Both models are widely adopted to test macroevolutionary hypotheses, which commonly requires constraining the parameters of the process, the origination and extinction rates. To this aim, statistical methods are necessary to generalize over realized rates seen in individual trajectories and to estimate the underlying rates of the common process generating these trajectories (Foote, 1988; Newman & Sibani, 1999). It should be noted that there are other methods available to estimate diversification rates which do not address commonalities in these rates within groups and thus avoid the problem of averaging the trajectories (Alroy, 2014; Foote, 2001; Silvestro et al., 2014; Sakamoto, Benton & Venditti, 2016; Liow & Finarelli, 2014). The approach using diversity trajectories is outlined here.

It is based on the intuition that analyzing a large set of diversity trajectories should reveal their shared properties. Therefore the symmetry emerging from averaging diversity trajectories across taxa has been interpreted as indication that both extinction and origination rates are equal and time-homogeneous (Quental & Marshall, 2013; Nee, 2006). From this, it also follows that rates calculated based on the averaged, symmetrical pattern have mirrored dynamics in the sense that the origination rate declines at the same pace as the extinction rate rises and the final value of one equals the initial value of another (see model A of Gilinsky & Bambach (1987) and Quental & Marshall (2013)). Moreover, the symmetric pattern has been used to argue that, at any moment in the Earth’s history, half of the taxa are diversifying and half are in decline (Quental & Marshall, 2010). There is, however, a discrepancy between the averaged pattern and the frequency of types of trajectories, for example individual symmetrical trajectories form only a marginal proportion of the datasets producing averaged symmetrical patterns (Quental & Marshall, 2013). The distribution of changes in the origination and extinction rates is skewed towards more common increases in the origination rates during the diversification phase of clades, although it is symmetrical during their decline phase. Similarly, origination declines are more common than extinction declines in diversity trajectories of marine taxa (Gilinsky & Bambach, 1987). This raises caution that the average symmetrical waxing and waning pattern does not allow us to conclude equal or time-homogeneous rates of the underlying branching process.

### Predicting extinction risk

Inferring age-dependent extinction risk is a question parallel to the age-area relationship discussed in the previous point; it is also among the questions where paleobiological data may aid conservation decisions. It has been shown across numerous clades and timescales that occupancy, geographic range and other MESs are among key predictors of extinction risk (Finnegan, Payne & Wang, 2008; Kiessling & Aberhan, 2007; Payne & Finnegan, 2007; Finnegan et al., 2015). Kiessling & Kocsis (2016) showed that the change in occupancy through time is a better predictor of extinction risk than standing occupancy. This brings into light the possibilities offered for conservation by the fossil record and the trajectories of species presence through time (Harnik et al., 2012). The potential of this approach can be compromised by averaging trajectories of the MESs across entire groups to predict extinction risk in higher level clades (Tietje & Kiessling, 2013).

### The way data are processed

The claim of this paper is directly linked to the way data are processed. We will refer to the following data-processing procedure to generate averaged trajectories:

1. Take the trajectories describing the presence of different taxa from their origination to their extinction
2. Shift each trajectory so the origination of the taxon is at time zero (x-shift)
3. Rescale each trajectory so the extinction of the taxon is at time one (x-scale)
4. Rescale the amplitude of all trajectories (y-scale)
5. Average all trajectories

**Figure 1:**
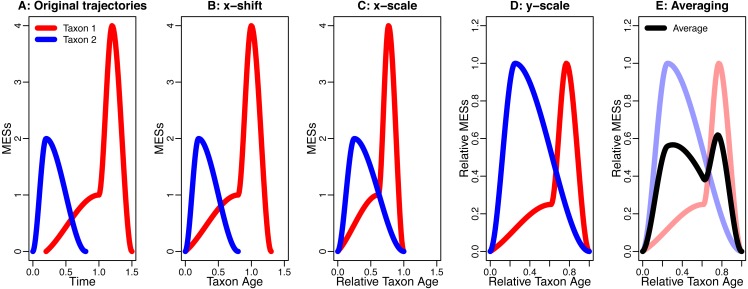
An example for the data processing procedure described in the section “The Way Data are Processed”. (A) Original trajectories; (B) x-shift; (C) x-scale; (D) y-scale; (E) Averaging. Note how the axis labels change due to the data processing.

This procedure is based on (Foote, 2007; Foote et al., 2007) and shown in Fig. 1.

One part of this data-processing procedure that will increase symmetry and similarity is averaging. This is demonstrated by Donsker’s invariance principle (Klenke, 2008, p. 474), which states that averaging different stochastic processes can lead to the same, symmetric stochastic process (section “Symmetry and Similarity by Averaging”). Another influence is the blurring effect of background noise that is being introduced into the analysis by the x-shift and the x-scale. This combines different sources of noise from different time intervals and of different taxa, which can drown possible signals (section “The Effect of Noise”). The last effect discussed here that can increase symmetry is conditioning. This describes the effect of already knowing the extinction and the origination, which puts an upper and a lower bound on the developments of the taxa in their lifespan, thereby making strongly asymmetric developments impossible (section “Symmetry by Conditioning”).

### Measuring symmetry

Symmetry and asymmetry are easily tangible by intuition. To make our results quantifiable and reproducible, we define a measure of asymmetry named QuAsy (Quantified Asymmetry).

A function *f* is axis symmetric (with respect to the y axis) if *f*(*x*) = *f*(−*x*). The symmetric part of said function is the largest symmetric function smaller than *f* and is given by *f*_*sym*_ = min{*f*(*x*), *f*(−*x*)}. The asymmetrical part of *f* is then given by *f*_*asym*_ = *f* − *f*_*sym*_, allowing to defined the QuAsy as

**Figure 2:**
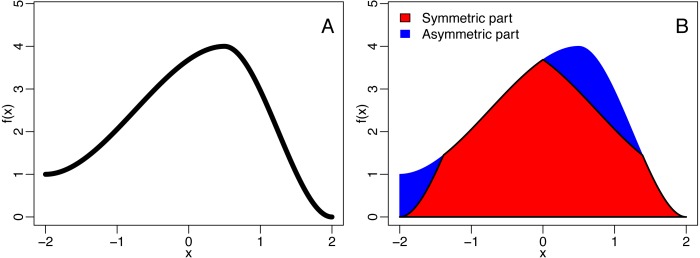
The decomposition of a function (A) into is symmetric and asymmetric part (B). The blue area corresponds to the quantified asymmetry (QuAsy) of the function introduced in the section “Measuring Asymmetry”.

(1) \begin{eqnarray*}{QuAsy}\nolimits (f)=\int \nolimits {f}_{asy}(x)\mathrm{d}x=\int \nolimits f(x)-\min \nolimits \{f(x),f(-x)\}\mathrm{d}x,\end{eqnarray*}

see Fig. 2 for an example. For binned data, the QuaSy is given by

(2) \begin{eqnarray*}{QuAsy}\nolimits (f)=\sum _{i=1}^{n} \left[ {f}_{i}-\min \nolimits \{{f}_{i};{f}_{n-i}\} \right] ,\end{eqnarray*}

where *f*_*i*_ is the value of the function in the *i*th bin. This measure of asymmetry is slightly weaker than a seminorm, and it is zero if and only if the function is symmetric. Its properties are described in Appendix S1.

## Three Ways to Increase symmetry and similarity

### Symmetry and similarity by averaging

One way to generate symmetry out of randomness and reduce distinguishability is averaging. Here, we take distinguishability as the ability to statistically distinguish hypotheses by the sets of trajectories generated and similarity as the opposite of distinguishability. The increase of symmetry and similarity by averaging is best known from the central limit theorem, which states that the averaged sum of random variables converges towards the symmetric standard normal distribution (Laplace, 1812). This is even the case for the sum of random variables with different distributions (Lindeberg, 1922). Similar results exist for stochastic processes. One such result is Donsker’s invariance principle (Klenke, 2008, p. 474)). It states that averaged sums of stochastic processes will converge to Brownian motion, a stochastic process that is commonly named the Wiener process in probability theory. Donsker’s invariance principle can be seen as an infinite dimensional version of the central limit theorem, with Brownian motion replacing the standard normal distribution. Just like the standard normal distribution, Brownian motion is highly symmetric:

• It is self-similar in the sense that rescaled versions of Brownian motion are again Brownian motion (Klenke, 2008, p. 455)
• It is time reversible in the sense that if (*W*_*t*_)_(*t*∈[0,1])_ is a Brownian motion process, then so is (*W*_1_ − *W*_1−*t*_)_(*t*∈[0,1])_.

Note that the reversibility of Brownian motion implies the axis symmetry of symmetric waxing and waning, as it is for example displayed in Fig. 2 of Foote (2007). This is because reversibility means that it is indistinguishable whether the measures of interest were recorded from origination to extinction or vice versa, which implies that the averaged trajectories look the same, independently of whether they are plotted from origination to extinction or from extinction to origination. In other words, averaging trajectories that are reversible results in symmetry along a vertical axis through the midpoint of the taxon’s life span. The idea of reversibility and its link to axis symmetry is further discussed in Appendix S2.

Donsker’s invariance principle is not only valid for a symmetric random walk, but also for the much broader class of stochastic processes with expectation value zero and finite variance (see Klenke (2008) p. 474 for technical details). Donsker’s invariance principle demonstrates two different effects of averaging:

1. Averaging increases similarity, since if averaged, different stochastic processes converge to the same limiting process
2. Averaging increases symmetry, since it can turn asymmetrical stochastic processes into symmetrical stochastic processes

**Figure 3:**
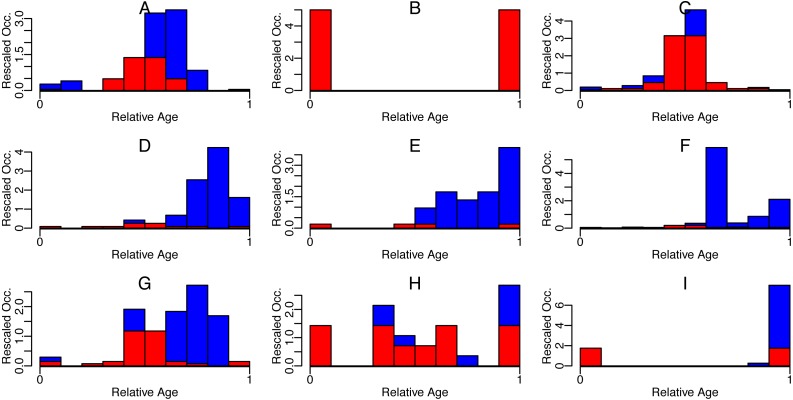
Symmetric (red) and asymmetric (blue) part of rescaled occurrences with relative age of some Cenozoic foraminifera species. Nine randomly selected species (A-I) are shown. Occurrences were downloaded from the Neptune Sandbox Berlin (NSB) (Lazarus, 1994; Spencer-Cervato, 1999), rescaled to have first and last occurrence at 0 and 1 resp. (x-shift and x-scale), and then binned into ten bins. The area of the bins was then rescaled to sum up to 1 (y-scale).

**Figure 4:**
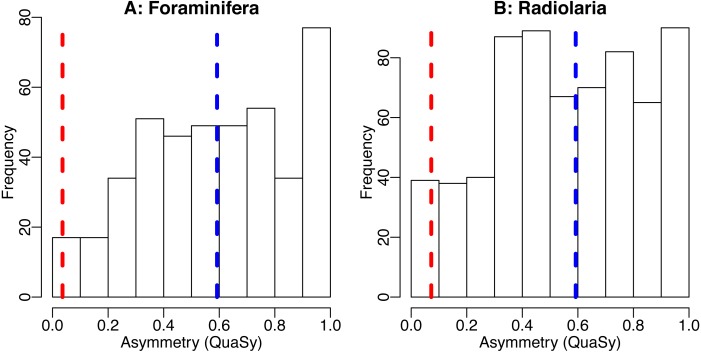
The distribution of asymmetry in occurrences of Cenozoic foraminifera (A) and radiolaria (B). Histogram shows the distribution of asymmetry of individiual species, average of the asymmetry of individual species (blue dashed line) and asymmetry of the trajectory generated by averaging the trajectories of all taxa (red dashed line). Asymmetry was quantified using QuAsy (see Appendix S1), where 0 corresponds to symmetry and 1 correspond to asymmetry. Data was downloaded from the Neptune Sandbox Berlin (NSB) (Lazarus, 1994; Spencer-Cervato, 1999), data processing is as described in the caption of Fig. 3 or in Appendix S3. The distribution of asymmetry for individual species displays a tendency towards asymmetry, with a mean QuAsy value of 0.59 for foraminifera and 0.56 for radiolarians. The asymmetry of the averaged trajectories of all taxa returns a much lower value of 0.036 for foraminifera and of 0.072 for radiolarians, a decrease of more than 85% in both groups. Only 4% of individual foraminifera taxa are more symmetric than the “average foraminifera” generated by averaging all taxa. The R code used can be accessed in the Supplementary Information.

So if the trajectories of any type of presence MESs of taxa are generated by some type of stochastic process, they will be affected by this averaging-induced increase in symmetry (see Figs. 3 and 4) and similarity.

It must be noted that Donsker’s invariance principle is based on the weak averaging factor $ \frac{1}{\sqrt{n}} $ as it is used in the central limit theorem, which guarantees that the result will be a stochastic process. Many limiting theorems, such as the weak and strong law of large numbers (Klenke, 2008, ch. 5), are based on the stronger averaging factor $ \frac{1}{n} $, making the limit a number, and not a distribution. Using this stronger averaging factor will therefore make the averaged trajectory smoother and emphasize the symmetry even more.

### The effect of noise

Another effect that reduces distinguishability is the combination of noise from different sources, which can make it hard to detect signals.

For example, occupancy trajectories of individual taxa are influenced by a variety of factors, like environmental conditions or biotic interactions, affecting a given taxon throughout its lifespan. Both the nature and timing of these factors may differ among taxa. By rescaling the trajectories of a group of taxa to start at zero and to end at one (x-shift and x-scale in the data processing procedure described in section “The Way Data are Processed”), signals that were originally in different time intervals are then placed in the same bin between zero and one. Analyzing these rescaled trajectories combines all the different signals from different sources and different effects on different taxa in different times. This leads to a strong background noise that can drown any potential signal.

As an example, take any real valued random variable *Y*_*n*_ describing the result of a statistical analysis after *n* samples have been evaluated. A common example for such a random variable is the sample mean, given by

(3) \begin{eqnarray*}{Y}_{n}= \frac{1}{n} \sum _{i=1}^{n}{X}_{i},\end{eqnarray*}

used as an estimator for the expectation value and as the basis for the construction of confidence intervals. In a more general setting, the task of *Y*_*n*_ can be to identify the underlying probability distribution by the realizations it generates.

Now, assume that the statistical analysis is well behaving in the sense that its quality increases with sample size and the corresponding random variable converges towards some deterministic value *a*. For applications, this raises the question about the number of samples required for the results of the statistical analysis to be reliable in the sense of displaying only fluctuations below some threshold around the limit *a*. We will show that the number of samples necessary for a reliable result increases as noise is introduced. This is formalized by adding perturbation random variables *Z*_*n*_ to the results of the statistical analysis. The new, perturbated analysis is described by the random variables

(4) \begin{eqnarray*}{\tilde {Y}}_{n}={Y}_{n}+{Z}_{n}.\end{eqnarray*}

In Appendix S2, we show that even in the best case scenario where the *Z*_*n*_ converge to zero, meaning that noise gets weaker as sample size increases, the perturbated random variables ${\tilde {Y}}_{n}$ fluctuate more than the original random variables *Y*_*n*_. This implies that the introduction of noise increases the number of samples necessary to obtain results of any chosen reliability. Conversely, for a fixed number of samples, results derived from a noisy scenario are less reliable than the ones from a scenario free of noise.

Note that no further assumptions on the type of statistical analysis, the type of noise or how they change with sample size were made.

### Symmetry by conditioning

Another effect that increases symmetry is the incorporation of the knowledge of the already known origination and extinction times of taxa. In probability theory, this is known as conditioning, and is based on the conditional probability. One example for conditioning can be found in Bayesian statistics, where the posterior is defined as the prior, conditioned on the observation. This incorporation of knowledge leads to a re-evaluation of the probabilities and the feasibility of trajectories, thereby putting boundary conditions on the trajectories of taxa:

• The MESs of a taxon cannot decline too much in the early part of its life, since otherwise it would go extinct
• The MESs of a taxon cannot increase too much in the second half of its life, since it has to go extinct at a fixed point

This leads to a necessary increase of the presence in the first half of a taxon’s life and a necessary decrease of the presence in the second half of a taxon’s life, which are both based on conditioning.

As an example for the effects of conditioning, we give a basic example using a random walk (*Y*_*n*_)_*n*∈ℕ_ (see Foote (2007),foote2014). The random walk is defined by

(5) \begin{eqnarray*}{Y}_{n}=\sum _{i=1}^{n}{X}_{i},\end{eqnarray*}

where

(6) \begin{eqnarray*}P({X}_{i}=1)=p\text{and}P({X}_{i}=-1)=1-p.\end{eqnarray*}

Here, *p* is a value between zero and one that we will call the upward transition probability and *X*_*i*_ is the change in position at the *i*th step. The random walk defined here is asymmetric in the sense that *p* can differ from 0.5 and is time-homogeneous in the sense that the transition probabilities do not change through time.

**Figure 5:**
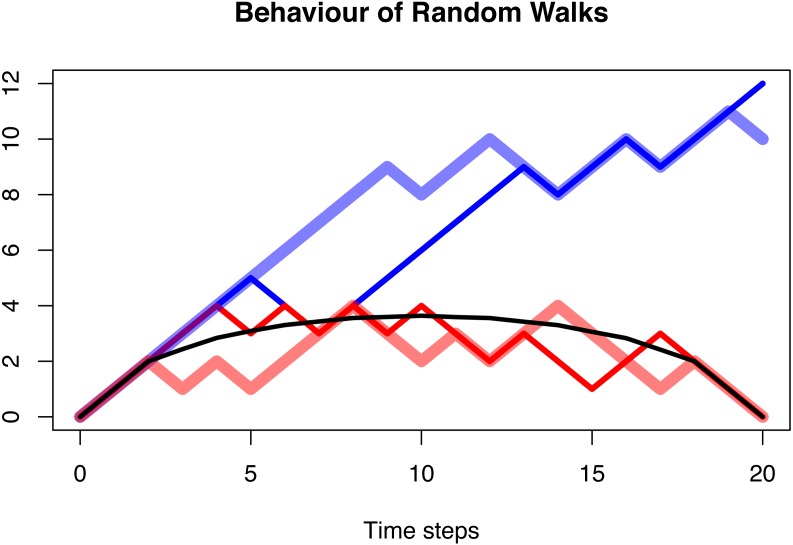
The behaviour of a random walk conditioned to be positive (blue), conditioned to be positive and go extinct after *n* = 20 time steps (red), and the average over multiple trajectories conditioned to be positive and go extinct (black). In all three cases, the probability of the random walk to increase is *p* = 0.6. The first case shows a clear increase as expected with a transition probability larger than 0.5. This is no longer visible in the second case, and the averaged and conditioned case is symmetric and lacks any random behaviour. The distribution of asymmetry of these three cases is shown in Fig. 3.

**Figure 6:**
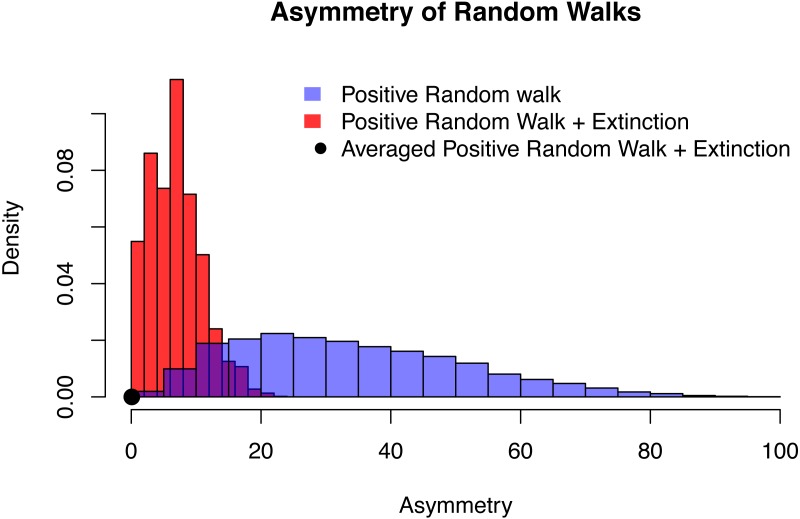
The distribution of asymmetry for an positive random walk (blue histogram), a positive random walk conditioned to go extinct (red histogram) and averaged trajectories of a positive random walk conditioned to go extinct (black dot). In all three cases, the probability of the random walk to increase is *p* = 0.6, time of extinction is after *n* = 20 time steps and the asymmetry was quantified using QuAsy (see Appendix S1), where 0 represents symmetry and high values correspond to high asymmetry. The distribution of asymmetry for the positive random walk going extinct is more concentrated and closer to zero than the distribution of the positive random walk, showing how conditioning increases symmetry. By additionally averaging the trajectories, this effect is emphasized even more. This is displayed by the averaged case (black dot) with a QuAsy value of below 0.1.

It can be shown that the trajectories of this random walk, conditioned on origination and extinction, are always symmetric after they have been averaged, independent of the choice of *p*. For the case *p* = 0.6, this is displayed in Figs. 5 and 6.

This result can be shown as follows: first, extinction can only occur every second time step. Second, it is sufficient to show that the averaged trajectories conditioned on extinction at 2*n* and origination at 0 are symmetric. This is because symmetry is not affected by x-scaling (see the section “The Way Data are Processed”).

For some fixed 2*n*, every path from origination at 0 to extinction at 2*n* consists of 2*n* steps. Since all feasible trajectories start with presence zero and end with presence zero, and the magnitude of change is the same in each step, half of the 2*n* steps must increase and half must decrease. Therefore every such trajectory has the probability *p*^*n*^(1 − *p*)^*n*^. If one of these trajectories is mirror symmetric, it will only contribute symmetrically to the averaged trajectory. If it is not mirror symmetric, it has a mirrored counterpart that has the same probability of occurring. So the asymmetry of this trajectory will in the long run be canceled out by the asymmetry of its mirrored counterpart. This shows that in the long run, all contributions to the averaged trajectory are either symmetric or will be balanced by their counterparts, making the averaged trajectory symmetric itself.

This demonstrates that for the model of an asymmetrical time-homogeneous random walk, conditioning makes it impossible to infer any information about *p* from the symmetry or asymmetry of the averaged trajectories. In Appendix S4.3, a more general line of argument shows that not only symmetry, but many properties derived from temporal trajectories will systematically change between the unconditioned and the conditioned case, which can lead to erroneous inferences (see Appendix S4.4).

## Implications for macroevolutionary studies

The three lines of argument in the section above show that

1. A large class of random processes will generate the same symmetric trajectories when they are averaged (section “Symmetry and Similarity by Averaging”)
2. Deviations from symmetry are hard to recognize in the presence of noise (section “The Effect of Noise”)
3. Fixing trajectories to predetermined times of origination and extinction increases symmetry (section “Symmetry by Conditioning”)

An immediate implication of these results is that the symmetric waxing and waning of the averaged trajectories, as it is for example observed in Foote (2007) and Foote et al. (2007) is not per se meaningful, since a large class of underlying processes is consistent with this pattern. Symmetric waxing and waning of the averaged trajectories should therefore not be used to test hypotheses of any kind, since it cannot be linked to the parameters of the underlying processes.

More generally, the three points above demonstrate that averaged trajectories do not need to reflect properties of the underlying processes, since they have an intrinsic tendency to increase symmetry and similarity. This reduces their sensitivity and thereby makes them unsuitable to derive information about the parameters driving the underlying process.

## Discussion

One of the central claims of this paper is that the shape of the trajectories per se is not of interest, but rather the information about the parameters of the underlying process that these shapes convey. Knowledge about the trajectories a model will generate and the ability to distinguish trajectories generated by different processes is therefore crucial to connect empirical observations and models.

The arguments presented in the section “Three Ways to Increase Symmetry and Similarity” show that averaged trajectories have an inherent tendency to increase symmetry as well as similarity and therefore cannot be linked to specific parameters of an underlying process. One conclusion from this is that symmetric waxing and waning as displayed in Foote (2007) and Foote et al. (2007) is unspecific and not uniquely linked to parameters driving an underlying process, as was demonstrated in the section “Symmetry by Conditioning”. We recommend abandoning the usage of averaged trajectories. A potential alternative is the nonparametric approach presented in Appendix S3. It avoids the problems arising from averaging and allows to non-parametrically test hypotheses regarding measures of eco-evolutionary success (MESs) of taxa against empirical data. The general framework provided by this approach also allows the application of multivariate methods.

There are statistical methods that do not rely on averaging (for example Liow & Finarelli (2014)), but the effects of noise and of conditioning are inherent to Paleobiology, where data is pooled over large time intervals and origination and extinction times of taxa are constrained. These two effects will be present independent of the statistical method used.

### Avoiding noise

In Appendix S3, we suggest a method that avoids averaging, one of the three effects discussed in this paper. This naturally raises the questions whether there are methods that avoid the other effects. The introduction of noise is directly linked to the combination of information from different time intervals and thereby unavoidable when questions about large scale processes are tackled. The chances are that this effect will lessen as both more data and higher resolutions of data are available in the future. That reduction of noise has an impact on results was demonstrated by Liow & Stenseth (2007), where a symmetric averaged trajectory becomes asymmetric as data with lower quality is removed.

### The generality of averaging

The effects of averaging can be strong and are not dependent on any model assumption. Especially two points contribute to this.

First, averaging condenses information about probability distributions. In descriptive statistics, this is a desired property, but information about important properties of the distribution such as skewness and the median are inevitably lost.

Second, averaging is deeply linked to most of the limit theorems in probability theory. What unifies all these limit theorems is the convergence of an average over some objects to an invariant object, which is not dependent on the properties of the objects that were averaged in the first place. Some famous examples of such invariant objects are the standard normal distribution (central limit theorem (Klenke, 2008, p. 319)(Lindeberg, 1922; Laplace, 1812)), Brownian motion (Donsker’s invariance principle, see the section “Symmetry and Similarity by Averaging” and (Klenke, 2008, p. 474)) or zero (weak and strong laws of large numbers (Klenke, 2008, p.108-119)). This tendency to converge to an invariant object will automatically make averages appear more similar and reduce their distinguishability.

The properties of averaging can also be passed down. This is for example displayed by the center of gravity (CG) defined by Gould, Gilinsky & German (1987). On average, it displays values that indicate symmetry. This is simply a reflection of the fact that the underlying averaged trajectories are symmetrical as a result of the points discussed in the section “Three Ways to Increase Symmetry and Similarity”.

### Conditioning of models

Conditioning formalizes the incorporation of additional knowledge into a model and is therefore common in paleontology, where potentially extinct taxa are examined. It alters the behaviour of a model by fundamentally changing the probability distribution that determine the probability of the temporal trajectories to be observed (Appendices S4.2 and S4.3). One example for this was given by Budd & Mann (2018), who showed that the analysis of extant clades favors clades with an early boost of diversification. This displays that in contrast to averaging (which is commonly part of a statistical procedure) conditioning is part of the question asked, making its effects independent of the statistical approach used.

By mathematical necessity, functions extracting information from trajectories will have a different expectation value for the conditioned and the unconditioned model (Appendices S4.3). Commonly a set of models is given and the task is to identify the model at hand based on the observed trajectories. The result above implies that trying to identify the unconditioned models based on conditioned trajectories will lead to misidentifications (Appendices S4.4, also containing an example).

For these reasons there will be no solution to avoid the effects of conditioning such as better data or new statistical methods. Conditioning completely changes the model used, and statistical methods must be adapted to this new model to avoid misinterpretations of paleontological data. This is especially important when both conditioned and unconditioned models are used, for example when geological data is used to predict developments in the near future.

## Conclusion

We show that averaging, background noise, and already pre-set extinction and origination times necessarily increase symmetry and the difficulty to statistically distinguish processes by means of the trajectories they generate.

The resulting symmetry of averaged histories of taxa is consistent with many potential scenarios, and does not allow to draw any conclusions about parameters of the underlying process, such as origination rates or extinction rates, or how these parameters change through time.

More general, the introduction of boundary conditions (conditioning) such as pre-set extinction and origination times fundamentally change the model used. Not adjusting the methodology to this new model will lead to erroneous inferences about the drivers of the temporal development of taxa.

## Publication History Statement

This manuscript is based on the preprint “Enforced Symmetry: The Necessity of Symmetric Waxing and Waning” (Hohmann, 2018).

## Data Accessibility Statement

The code used for the examples and figures was written in R Core Team (2015) and can be accessed in the supplementary materials, the raw data used is deposited on the Open Science Framework (OSF) and can be downloaded under http://osf.io/zw5ef/.

##  Supplemental Information

10.7717/peerj.8011/supp-1 Supplemental Information 1 R code used for the figures and examples in the appendix

10.7717/peerj.8011/supp-2 Supplemental Information 2 Appendix containing (1) a description of the method used to quantify asymmetry (2) mathematical formalizations of the lines of argument made in the text and (3) potential solutions to the problem arising from averaging data

## Additional Information and Declarations

### Data Availability

The following information was supplied regarding data availability: The code used for the examples and figures was written in R (version 3.2.3) and is available in the Supplemental Files. The raw data is available at the Open Science Framework (OSF): Hohmann, Niklas. 2019. “Raw Data: Enforced Symmetry: The Necessity of Symmetric Waxing and Waning.” OSF. October 15. doi:10.17605/OSF.IO/F8YHQ.
