# Supplementary material for: Enforced symmetry: the necessity of symmetric waxing and waning
Source: PeerJ. 2019 Nov 6;7:e8011. doi: 10.7717/peerj.8011 (PMC6842294; doi:10.7717/peerj.8011)
Supplement: Supplemental Information 2 [file peerj-07-8011-s002.pdf]

Enforced Symmetry: The Necessity of Symmetric Waxing and Waning: **Appendices**

Niklas Hohmann* Emilia Jarochowska

1 Measuring Asymmetry

The asymmetry of a function f can be measured by decomposing it into a symmetric and an asymmetric part (see fig. 1), which can then be quantified using a seminorm on the underlying function space. The proposed concept is more sensitive as a measure of symmetry than the center of gravity used by Gould et al. (1987) in the sense that it is zero if and only if the corresponding function is axis symmetric, whereas a balanced center of gravity is not a necessary condition for symmetry.

A function g is mirror symmetric (short symmetric) with respect to the y-axis if $g(x) = g(-x)$. A function g is mirror symmetric with respect to the axis defined by $x = a$ if the function $g(x-a)$ is symmetric with respect to the y-axis. For simplicity we only examine the first case, the second case follows mutatis mutandis.

For any function f, its symmetric part is defined as

$$f_{sym}(x) = \min\{f(x), f(-x)\} \ . \tag{1}$$

It is maximal in the sense that it is the biggest symmetric function that is smaller than f. The asymmetric part of f is given by

$$f_{asy}(x) = f(x) - f_{sym}(x) \ . \tag{2}$$

*corresponding author, email: Niklas.Hohmann@fau.de

Let

$$\text{asy}\colon f \mapsto f_{asy} \tag{3}$$

be the operator that assigns every function f its asymmetric part, so $\text{asy}(f) = f_{asy}$. It has the following properties:

$$\text{asy}(f) \equiv 0 \iff f \text{ is symmetric} \tag{4}$$

$$\text{asy}(\lambda f) = \lambda\,\text{asy}(f) \text{ for } \lambda \geq 0 \quad \text{(positive homogeneity)} \tag{5}$$

$$\text{asy}(f+g) = \text{asy}(f_{asy} + g_{asy}) \leq \text{asy}(f) + \text{asy}(g) \quad \text{(sublinearity)}\ . \tag{6}$$

Combining this operator with any monotonous seminorm $\|\cdot\|$ on the vector space of functions yields the function

$$\|f\|_{asy} := \|\,\text{asy}(f)\| \tag{7}$$

measuring the degree of asymmetry of the function f. We will refer to $\|f\|_{asy}$ as quantified asymmetry, short QuAsy. It is slightly weaker than a seminorm in the sense that it is only positively homogeneous and not absolutely homogeneous.[1]

As an example, taking vectors in $\mathbb{R}^n$ as functions and using the 1-norm

$$\|x\|_1^d = \sum_{i=1}^{n} |x_i| \tag{8}$$

yields the QuAsy

$$\|x\|_{asy} = \sum_{i=1}^{n} |x_i - \min\{x_i; x_{n-i}\}| \tag{9}$$

measuring the asymmetry of binned data. Its continuous equivalent can be obtained by using the norm

$$\|f\|_1^c = \int |f(x)|\mathrm{d}x \tag{10}$$

instead of $\|\cdot\|_1^d$.

[1]Note that the term “positively homogeneous” is not used consistently in the literature. Here we use it in the sense of equation (5)

2 The Effect of Noise

Here, the example from the section "The Effect of Noise" is formalized and the stated result is derived formally.

By the assumptions made in the example in the main text, the real valued random variables Y_n describing the result of a statistical analysis after n samples have been evaluated converge to some deterministic value a and the random variable Z_n describing the contribution of the noise converge to 0. Let P_n be the distributions of Y_n and Q_n the distributions of the Z_n. Adding random variables is equivalent to convoluting their distributions, so $\tilde{Y}_n$ has the distribution $P_n * Q_n$, where $*$ denotes the convolution (Klenke, 2008, p. 277).

We will show that $P_n * Q_n(A) \gtrapprox P_n(A)$ as $n \to \infty$, meaning that the perturbed analysis is in the long run more likely to show results in any set A than the original analysis. Applying this to any set that does not contain a shows that the probability of deviations from the value a are higher in the perturbed analysis than in the original analysis.

The main result of the theory of large deviations roughly states that

$$\lim_{n\to\infty} P_n(A) \approx \exp(-n \inf_{z\in A} I(z)) \tag{11}$$

and

$$\lim_{n\to\infty} P_n * Q_n(A) \approx \exp(-n \inf_{z\in A} J(z)) \tag{12}$$

for two so called rate functions I, J that determine the rate of decay of the probability of the set A as n increases (Klenke, 2008, ch. 23) (Varadhan, 1984). To show the desired inequality, it is therefore sufficient to show that $J(z) \leq I(z)$ for all z, meaning that $P_n * Q_n$ decays slower than P_n.

To show this, let P_n and Q_n satisfy a large deviation principle (LDP) with rate functions F_P and F_Q[2]. By the assumption on the convergence of the noise, we have $F_Q(0) = 0$, and by shifting the P_n

[2] The existence of a LDP is not a strong assumption, since LDPs are known in many cases (e.g. for the Brownian motion, empirical measures, averages of i.i.d. random variables) and preserved under a number of operations, e.g.

by a, we can without loss of generality assume that $F_P(0) = 0$. Then the product measures $(P_n \otimes Q_n)_{n \in \mathbb{B}}$ on $\mathbb{R}^2$ satisfy a LDP with rate function $R(x, y) = F_P(x) + F_Q(y)$ (under the assumption that both P_n and Q_n are exponentially tight) (Kühn, 2014, lemma 2.7). The image measures of $(P_n \otimes Q_n)$ under the function $f(x, y) = x$ are the P_n, which do, according to the contraction principle (see (Klenke, 2008, p. 518)) and by definition, satisfy a LDP with rate function $I(z) = F_P(z)$.

Next, take the function $g(x, y) = x + y$. The image measure of $(P_n \otimes Q_n)$ under this function is $P_n * Q_n$ by the definition of the convolution. Applying the contraction principle yields the rate function

$$J(z) = \inf_{x+y=z} [F_P(x) + F_Q(y)] \tag{13}$$

for the LDP of $P_n * Q_n$. By setting $x = z, y = 0$ and $x = 0, y = z$, the inequality

$$J(z) \leq \min\{F_P(z), F_Q(z)\} \tag{14}$$

follows. Although this estimate is not very elaborate and can certainly be improved, it is enough to show that

$$J(z) \leq \min\{F_P(z), F_Q(z)\} \leq F_P(z) = I(z) \tag{15}$$

which is the desired statement.

3 Avoiding Averaging

To avoid averaging, we propose a nonparametric approach that can be used to test hypotheses about the processes that underlie the measures of eco-evolutionary success (MESs) of taxa throughout their life.

The approach exploits the fact that although the taxon's MESs are in theory continuous in time, the way geological time is resolved imposes that they are, in most cases, described by assigning records of a taxon into a finite number of time bins. A MESs is then given by n bins, with

pushforward measures under continuous functions, formation of product spaces and projective limits. This allows for a variety of constructions.

each bin assigned a number describing the MESs of the taxon in the corresponding time interval. These bins can be taken as components of a vector, where the value of the k-th bin is the value in the k-th entry of the vector. This shifts the problem of recognizing patterns in the temporal trajectories from time series analysis to multivariate statistics while preserving the structure of the data as autocorrelated time series since the components of the vectors remain ordered and correlated.

Multivariate statistics provides tests to decide whether two sets of points (time series) were sampled from the same distribution or not. Here we will focus on the multivariate Cramér test (Baringhaus and Franz, 2004; Cramer, 1928), a test whose univariate version is closely related to the Kolmogorov-Smirnoff test. In the context of the problem posed in this paper, it can be used to decide whether the trajectories of two sets of taxa were generated by the same underlying process or not, even if no knowledge of the nature of that underlying process is available. The test can further be used to test a model against empirical data. For this, temporal trajectories are simulated according to the model, binned into n bins and transformed into an n-dimensional space. Comparing the distribution of the point clouds of the simulated trajectories and the empirical trajectories provides information about the plausibility of the model. This procedure is demonstrated in example 1 by testing the the age-area hypothesis from Willis (1922).

The described approach can also be used to create a nonparametric, distribution-free test for symmetry. For this, we propose to replace the notion of symmetry of the averaged trajectories by reversibility in time of the trajectories, a concept common in time series analysis (Lawrance, 1991). Reversibility is more sensitive than symmetry of the averaged trajectories in the sense that reversibility implies symmetry of the averaged trajectories, whereas the converse is not true. Using reversibility is motivated by the property of the Brownian motion mentioned in the section "Symmetry and Similarity by Averaging" and by Gould et al. (1987), referring to the statement of Morris (1984) "that the world has a different appearance in one direction of time than it does in the other" if there is such a thing as an "arrow of time". In a reversible process, every asymmetrical trajectory is at some point balanced by the appearance of its mirrored version. An example

of the procedure to test reversibility can be found in example 2.

For simplicity, the number of occurrences is used as a proxy for abundance in both examples. All calculations were done using R (R, version 3.2.3), the commented code is attached. The raw data used for the examples is accessible via the Open Science Framework (OSF) under osf.io/zw5ef/.

3.1 Example 1: Testing the Age-Area Hypothesis

The age-area hypothesis as formulated by Willis (1922) has been rejected as a general ecological pattern (Gaston, 1998) and only serves as a well-known example to demonstrate the described approach. We test a reformulation of it, which states that taxa have a constantly increasing abundance throughout their life. The idea behind testing this hypothesis was outlined above and can be generalized to any other hypothesis about the development of MESs throughout the life of taxa.

3.1.1 The Data

As the empirical data, marine microfossil data from the Neptune Sandbox Berlin (NSB) (Lazarus, 1994; Spencer, 1999) was used. The data was downloaded on September 02, 2018 and contains information about occurrences of foraminifera. Questionable identifications, taxa invalidly included in the fossil group, open-nomenclature taxa, problematic samples, and problematic occurrences were filtered out. The taxonomy was resolved using the Taxonomic Name List (TNL) project of the IODP (Renaudie et al., 2015), the age model used is based on Gradstein et al. (2012). The age of the samples was restricted to the interval between 1 and 65.5 Ma to ensure comparability with Liow and Stensteth (2007). After removing species with only one occurrence, the dataset contained 108882 occurrences from 452 species, with a mean of 254 occurrences per species and a median of 76 occurrences per species. For each species, the ages were rescaled for the first occurrence to be at 0 and the last occurrence at 1 and then binned into $n = 10$ bins of equal duration. Last, for each species, the values of the bins were rescaled for the combined area of the bins to have an area of one. This makes sure that every species has the same contribution

to the analysis, independent of its number of occurrences.

3.1.2 Creating the Distribution of the Age-Area Hypothesis

The trajectories representing the age-area hypothesis were generated in a stochastic model that assumes that although the abundance of taxa is constantly increasing, it is still subject to random fluctuations.

The presence p of a taxon at time t is assumed to follow the equation

$$a(t) = mt + W_t \tag{16}$$

where m is a positive number and W_t is a Brownian motion. The parameter m determines how strong the expansion of the taxon is and was set to $m = 5$ for the simulation. A taxon originates at $t = 0$ and will go extinct if (1) its trajectory hits zero due to the fluctuations of the Brownian motion or (2) it survives until time $t = 1$. In the first case, the trajectory is rescaled to go extinct at $t = 1$ in accordance with the procedure described in the section "The Way Data is Processed". The number of trajectories simulated is identical to the number of taxa in the empirical data. Each trajectory was binned with the bins used above, the value of the bin with borders t_i, t_{i+1} is given by

$$b_i = \int_{t_i}^{t_{i+1}} a(t)\mathrm{d}t \tag{17}$$

Last, for each trajectory, the values of the bins were rescaled for the combined area of the bins to have an area of one to make them comparable with the bins from the dataset given above.

3.1.3 Testing the Hypothesis

The procedure described above generated two sets of points in a ten-dimensional space: One set for the simulated trajectories, and one derived from the Neptune database. Each point within these sets represents the trajectory of one taxon. Applying the multivariate Cramér test for the two sample problem (Baringhaus and Franz, 2004) as implemented in the R package "cramer"

(Franz, 2014) to these two datasets of points returns that the hypothesis of equal distribution of both datasets should be not be accepted ($p = 0$). Therefore the age-area hypothesis is rejected.

3.2 *Example 2: Testing Reversibility*

Testing reversibility is based on the fact that reversing a binned trajectory is equivalent to reversing the order of the entries of the corresponding vector. So to test invariance under time reversal, a sample is divided into two subsamples, one of which is reversed. If both subsamples have the same distribution, the original sample is reversible (Lawrance, 1991). To make sure this is not dependent on the division into subsamples, the test should be repeated multiple times with subsamples randomly drawn from the original sample

To demonstrate this test, radiolarian data was downloaded from the NSB database (Lazarus, 1994; Spencer, 1999) using the same parameters, options and rescaling as described above. Overall, there were 97811 occurrences from 667 species, with the median of the number of occurrences per species being 48 and the mean 146.6 . For every species, the rescaled ages were binned into $n = 10$ bins. The bins of each species were then rescaled to have an area that sums up to one. Then two sets were created: one with the species whose histories were reversed in time and one with those whose trajectories were left unchanged. Each species was randomly assigned to one of these groups with a probability of 0.5. For these two datasets, the multivariate Cramér test for the two sample problem (Baringhaus and Franz, 2004) as implemented in the R package "cramer" (Franz, 2014; R, version 3.2.3) was used to compare whether they were generated by the same distribution. The test was repeated 1000 times, each time with newly assigned unchanged and reversed datasets. In all of the 1000 runs, the hypothesis of equal distribution was rejected. The median over all p-values was 0, the mean 0.00045 and the maximum 0.003996 (see fig. 2). Therefore the hypothesis that the underlying distribution is invariant under time reversal is rejected. This suggests that the temporal dynamic of occurrence frequency observed among radiolarians is inconsistent with the effects of a reversible stochastic process, although the results from Liow and Stensteth (2007) suggest otherwise.

4 The Effects of Conditioning

4.1 *Formalizing Conditioning*

In the following, we will call some space $E \times A$ combined with a probability distribution P a model. The set E will represent the part of the space on which the conditioning will take place. In the cases discussed in the paper, E is the set of all values of temporal trajectories at their temporal endpoints and A is the set of all temporal trajectories without their last value. The order of E and A was changed for consistency of notation with the mathematical literature. The probability measure P determines the probability of any temporal trajectory to appear. By theorem 1.23 in Kallenberg (2017), P can be decomposed into a Markov kernel (transition kernel) Q from E to A and a probability distribution p on E such that $p \otimes Q = P$. Here p describes the probability of the trajectories (in the unconditioned model) to arrive at a given value at the temporal endpoint and $\otimes$ is the product of the probability measure and the kernel. This decomposition is characterized via

$$\int_{E\times A} f(e,a)\ P(\mathrm{d}e,\mathrm{d}a) = \int_{E\times A} f(e,a)\ p\otimes Q(\mathrm{d}e,\mathrm{d}a) = \int_E \int_A f(e,a)\ Q_e(\mathrm{d}a)p(\mathrm{d}e) \tag{18}$$

Now chose any probability distribution p' on E. The model conditioned to have the probability distribution p' on E is then given as the product $p' \otimes Q$. Conditioning of the model to deterministically end at some value $i \in E$ is formalized by setting $p' = \delta_i$, where δ_i is the Dirac measure on i.

4.2 *Uniqueness of Probability Distributions*

Probability distributions are uniquely determined by the integrals they define in the sense that if

$$\int f(x)\ P_1(\mathrm{d}x) = \int f(x)\ P_2(\mathrm{d}x) \tag{19}$$

for all f from some class of functions $\mathcal{F}$, then $P_1 = P_2$. Important classes of functions are continuous functions with compact support and bounded continuous functions ((Klenke, 2008),

chapters 13.1 and 15.1). By this statement, the definition of the product $\otimes$ and the construction of the conditioned models we obtain that $p_1 \otimes Q \neq p_2 \otimes Q$ if $p_1 \neq p_2$. So conditioned models are only identical if they are based on the same probability distribution on E.

4.3 *Systematic Differences Among Conditioned Models*

The characterization in the subsection above implies that for a large number of functions, integrals over these functions yield different results for different probability measures. Translating this into the dialect of probability theory by replacing the integral $\int f(x)\ P(\mathrm{d}x)$ by the expectation value $\mathrm{E}_P[f(X)]$ shows that the expectation value of many functions that quantify a property of temporal trajectories will differ from the unconditioned model to the conditioned model. As an example for the systematic differences between conditioned models and unconditioned models, assume that E is finite and that without loss of generality $E = \{0, 1, \ldots, N\}$. Then by the definition of the product of kernels (see Kallenberg (2017), lemma 1.17) for any function f on $E \times A$ we obtain

$$\int f(e,a)\ P(\mathrm{d}e, \mathrm{d}a) = \int f(e,a)\ p \otimes Q(\mathrm{d}e, \mathrm{d}a) = \sum_{i=1}^{N} p(i) \int f(e,a)\ \delta_i \otimes Q_i(de, da) \tag{20}$$

where $p(i)$ is the probability of the unconditioned model that a trajectory ends at i and $\delta_i \otimes Q_i(de, da)$ is the probability distribution describing the model conditioned to end at value i. Similarly if the model is conditioned to end with probability distribution q, we obtain

$$\int f(e,a)\ q \otimes Q(\mathrm{d}e, \mathrm{d}a) = \sum_{i=1}^{N} q(i) \int f(e,a)\ \delta_i \otimes Q_i(de, da) \tag{21}$$

This shows that every conditioned model is a convex combination of the models that deterministically end with value $i \in E$. By defining the simplex

$$\Delta := \{x \in \mathbb{R}^N \mid x_i \geq 0 \text{ and } \sum x_i = 1\}\ , \tag{22}$$

every conditioned model can be uniquely identified by the mapping

$$x \mapsto \sum_{i=1}^{N} x_i\ \delta_i \otimes Q_i \tag{23}$$

where $x \in \Delta$. Accordingly we get

$$\int f(e,a)\tilde{p} \otimes Q(\mathrm{d}e, \mathrm{d}a) = \sum_{i=1}^{N} x_i a_i \tag{24}$$

where $a_i = \int f(e,a)\delta_i \otimes Q(\mathrm{d}e, \mathrm{d}a) \in \mathbb{R}$ and $x_i = q(i)$. Maximizing (minimizing) the integral for a fixed function f and varying conditioned models is therefore equivalent to maximizing (minimizing) the linear function $\sum_{i=1}^{N} x_i a_i$ over Δ. This is a linear optimization problem, therefore its optima can be found in the vertices of the simplex, which represent the models conditioned to deterministically end at some value.

This abstract example becomes more alive when the elements of E are taken as the number of species living in the present day. It then shows that no matter what inference is made from the temporal development of species richness, it might be biased in both directions dependent on how well we know the species richness in the present.

4.4 Identifying Models

Take any set of models $(P_l)_{l \in L}$, indexed by a set L. Let

$$\mathcal{L} \colon E \times A \to L \tag{25}$$

be a function that tries to identify the model at hand based on the observed trajectories. Its expectation value (under an abuse of mathematical dialect), given that the model $P_{l'}$ is present is given by

$$\int \mathcal{L}(e,a)\ P_{l'}(\mathrm{d}e, \mathrm{d}a) \tag{26}$$

Assume that the function $\mathcal{L}$ does a good job in identidying the model in the sense that its expectation value is close to l' if the model $P_{l'}$ is assumed.

Now transition to the conditioned models derived from $P_{l'}$, here denoted by $\delta_j \otimes Q^{l'}$ for $j \in E$. Then by the line of argument in the subsection above, $\int \mathcal{L}(e,a)\ \delta_j \otimes Q^{l'}(\mathrm{d}e, \mathrm{d}a)$ will differ from l'. So trying to identify unconditioned models on the basis of data derived from conditioned models will lead to the misidentification of the models.

As an example of this effect take the random walk models defined in the section ”The Effect of Noise”. The different unconditioned models can be uniquely identified by their transition probability p, which can be estimated by $\frac{1}{2} + \frac{1}{2}\overline{X}$, where $\overline{X}$ is the mean of the X_i. However for the models conditioned to go extinct, the sum of the increasing and decreasing steps must be zero, so the upper estimator will always return the deterministic estimate $\frac{1}{2}$ independent of (1) the value of p of the random walk model conditioned in the first place (2) the time of extinction used for the conditioning and (3) the path taken by the random walk.

5 Figures

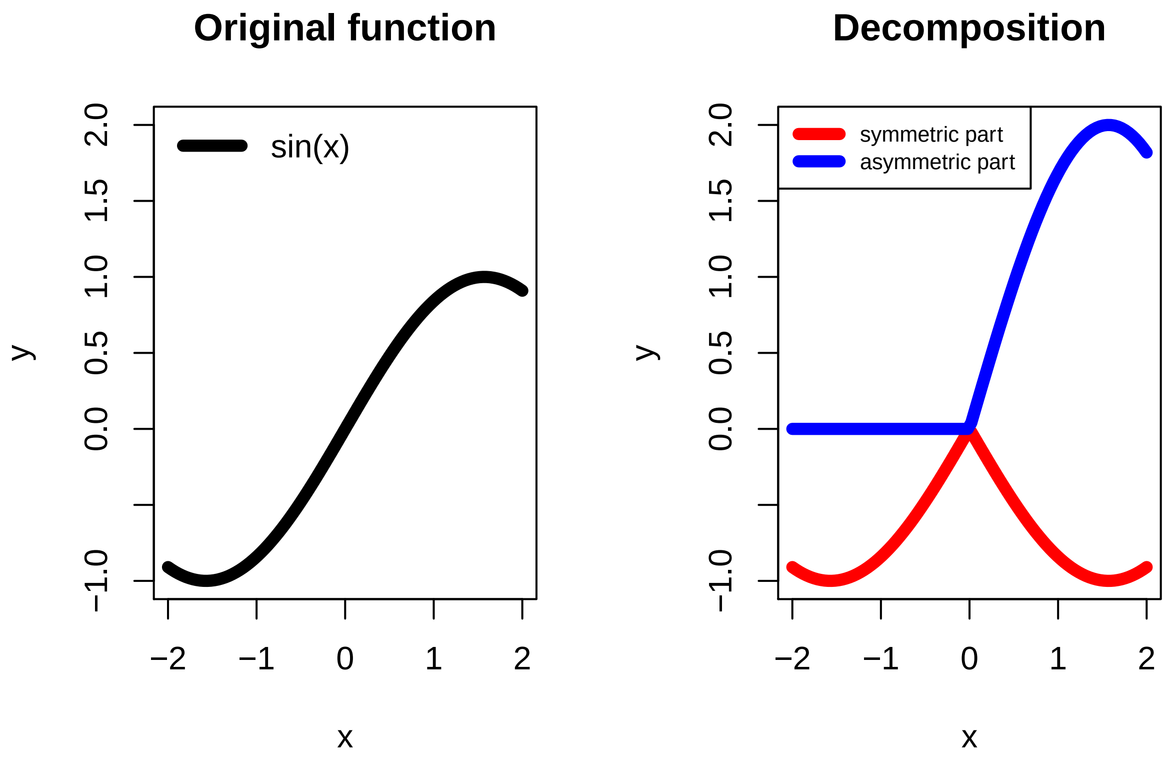

Figure 1: The decomposition of the function $f(x) = \sin(x)$ (left) into its symmetric part (right, red line) and its asymmetric part (right, blue line). Adding the asymmetric and the symmetric part yields the original function.

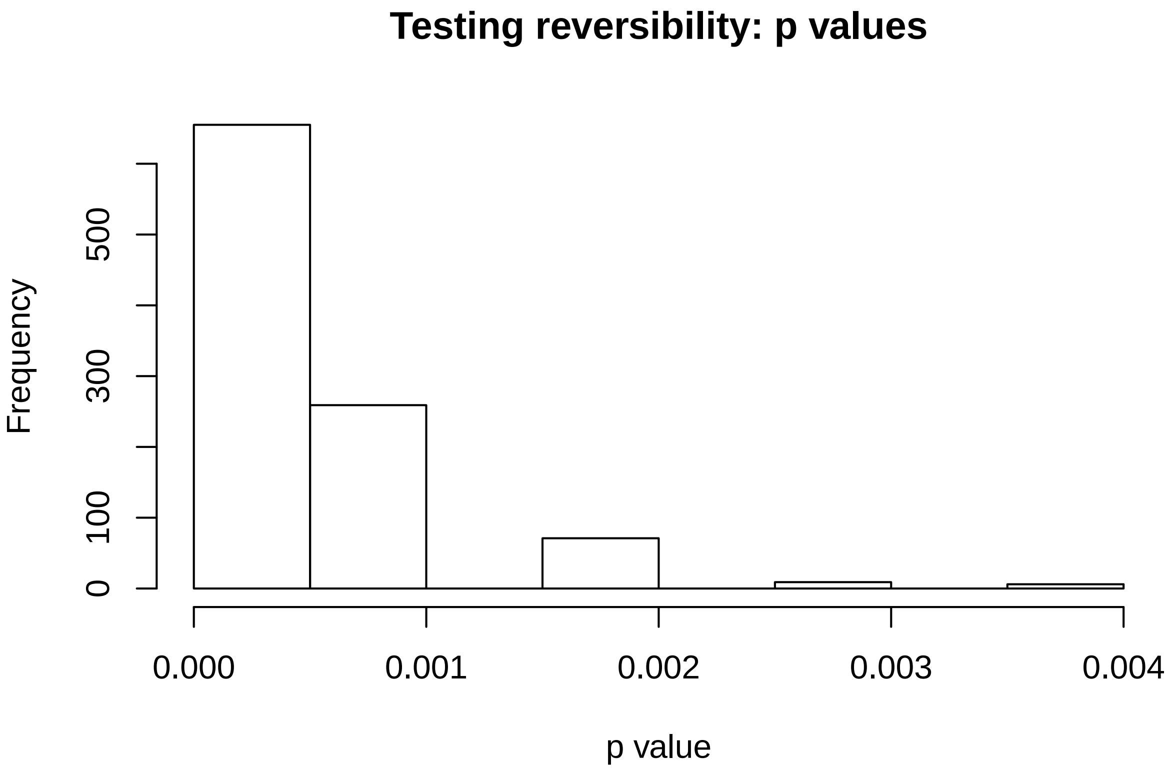

Figure 2: The distribution for the p values of the test for reversibility. Overall 1000 tests were performed.

References

Baringhaus, L., and C. Franz. 2004. On a new multivariate two-sample test. Journal of Multivariate analysis 88(1):190–206.

Cramér, H. 1928. On the composition of elementary errors: Second paper: Statistical applications. Scandinavian Actuarial Journal 1928(1):141–180.

Franz, C. 2014. cramer: Multivariate nonparametric Cramer-Test for the two-sample-problem R package version 0.9-1, https://CRAN.R-project.org/package=cramer .

Gaston, K. J. 1998. Species-range size distributions: Products of speciation, extinction and transformation. Philosophical Transactions of the Royal Society of London B: Biological Sciences 353(1366):219–230.

Gould, S. J., N. L. Gilinsky, and R. Z. German. 1987. Asymmetry of lineages and the direction of evolutionary time. Science 236(4807):1437–1441.

Gradstein, F. M., J. G. Ogg, M. D. Schmitz, and G. M. Ogg. 2012. The geologic time scale. Boston: Elsevier.

Kallenberg, O. 2017. Random Measures. New York: Springer.

Klenke, A. 2008. Probability Theory. A Comprehensive Course. London: Springer.

Kühn, F. 2014. Large Deviations for Lévy(-Type)Processes. Diploma thesis, TU Dresden.

Lawrance, A. J. 1991. Directionality and Reversibility in Time Series. International Statistical Review / Revue Internationale de Statistique, 59(1):67–79

Lazarus, D. 1994. Neptune: A marine micropaleontology database. Mathematical Geology 26(7):817–832.

Liow, L. H., and N. C. Stenseth. 2007. The rise and fall of species: Implications for macroevolutionary and macroecological studies. Proceedings of the Royal Society of London B: Biological Sciences 274(1626):2745–2752.

Morris, R. 1984. Time's arrow. New York: Simon and Schuster.

R Core Team. 2015. R: A Language and Environment for Statistical Computing. Vienna, Austria, 2015: R Foundation for Statistical Computing.

Renaudie, J., P. Diver, and D. Lazarus. 2015. Nsb: A new, expanded and improved database of marine planktonic microfossil data, 11 2015. Conference paper for the GSA Annual Meeting in Baltimore 41:1

Spencer-Cervato, C. 1999. The cenozoic deep sea microfossil record: Explorations of the dsdp/odp sample set using the neptune database. Palaeontologia electronica 2(2):270.

Varadhan, S.R.S. 1984. Large Deviations and Applications Philadelphia, PA: Society for industrial and applied mathematics.

Willis, J. C. 1922. Age and area. Cambridge: The University Press.
